# Evaluation of large language models in rheumatology and clinical immunology: a systematic assessment based on Chinese national health professional qualification examination

**DOI:** 10.3389/fmed.2025.1716122

**Published:** 2026-01-15

**Authors:** Yaqing Wang, Yue Jiang, Wen Jin, Yijun Xu, Weinan Lin, Jiangda Wang, Qin Song, Zhaoxi Fang

**Affiliations:** 1 Department of Transfusion Medicine, The Affiliated Hospital of Shaoxing University, Shaoxing, China; 2 Department of Computer Science and Engineering, Shaoxing University, Shaoxing, China; 3 Institute of Artificial Intelligence, Shaoxing University, Shaoxing, China; 4 Zhejiang Pharmaceutical University, Ningbo, Zhejiang, China; 5 School of Computing, College of Science, Engineering and Technology, The University of South Africa, Roodepoort, South Africa; 6 Zhejiang-ltaly Joint Laboratory on AI & Materials Medical Technology, Shaoxing, China

**Keywords:** DeepSeek, immunology, knowledge test, large language models, model evaluation

## Abstract

In recent years, large language models (LLMs) have achieved remarkable progress in natural language processing and demonstrated potential applications in medicine. However, their professional capabilities in specific medical subfields, such as immunology, still require systematic evaluation. This study systematically evaluated 11 representative LLMs, including DeepSeek, GPT, Llama, Gemma, and Qwen series, based on the Chinese National Health Professional Qualification Examination in Rheumatology and Clinical Immunology. The evaluation covered four dimensions: basic medical knowledge, related medical knowledge, immunology knowledge, and professional practice ability. Results show significant differences among LLMs. DeepSeek-R1 and Qwen3 achieve the best performance, with accuracy exceeding 90%. However, performance on professional practice ability tasks remained relatively low, highlighting limitations in complex clinical applications.

## 1 Introduction

Since OpenAI released the generative pre-trained Transformer model in 2018, large language models (LLMs) have experienced rapid development (1). Models such as GPT-3 and GPT-4 have demonstrated outstanding performance in numerous natural language processing tasks due to their powerful language understanding and generation capabilities (2). Concurrently, multiple research institutions and companies worldwide have launched distinctive models, such as the DeepSeek series (3), Google's Gemma series (4), Meta's Llama series (5), and Alibaba's Qwen series (6). These models vary in parameter scale, architecture design, and training data, fostering prosperity and competition in the field of artificial intelligence.

Explorations into the application of LLMs in the medical field are increasing, covering areas such as medical question answering, auxiliary diagnosis, medical record summarization, and patient education (7–10). For example, Google's Med-PaLM 2 was the first to achieve a score above the passing threshold of 86.5% on the United States Medical Licensing Examination (USMLE), indicating that LLMs possess clinically actionable value in medical diagnostic scenarios (11). In specific fields such as mental health, studies have begun systematically evaluating LLMs' knowledge mastery and diagnostic capabilities (12, 13). In (13), the authors tested the professional knowledge of LLMs like DeepSeek, Gemma, and GPT in mental health. The results showed that the highest accuracy on single-choice questions reached 86.83%, but the accuracy was lower on multiple-choice questions. In radiology error detection and case-based question answering, GPT-4V's performance approached that of radiologists; preliminary studies in pathological image-assisted diagnosis also show potential (14).

As an important subfield of medicine, LLMs also have broad application prospects in immunology (15–18). Rider et al. evaluated the diagnostic and clinical management guidance capabilities of LLMs in real cases of primary immunodeficiency disorders (15). In the study, real anonymized cases were submitted to the models using multi-turn prompting, and immunology experts assessed the models' diagnostic accuracy and reasoning quality. They found that some models (e.g., GPT-4o) achieved diagnostic accuracy of approximately 96.2%. In (16), researchers used LLMs to automatically identify immune-related adverse events in electronic health records (EHRs) and clinical trial data during treatments such as immune checkpoint inhibitors. This system can help detect these side effects in vast amounts of unstructured text, improving safety monitoring. In (17), researchers attempted to use GPT-4 as a tool for estimating the proportions of various immune cell types in mixed samples from whole blood RNA-Seq data. The results showed approximately 70% consistency between GPT-4 and traditional bioinformatics tools (e.g., CIBERSORTx, xCell).

Although LLMs demonstrate strong application potential in the medical field, their professionalism, accuracy, and reliability still require rigorous evaluation and validation. Currently, evaluations of LLMs' medical application capabilities mostly focus on general medical knowledge or a few popular specialties (13, 19). Evaluation methods include using standardized medical examination question banks (e.g., USMLE) (20), constructing specific task datasets (21), or simulating clinical scenarios (22). Only a small number of works have attempted to evaluate LLMs' capabilities in the field of immunology, such as (15). However, these works only evaluated specific tasks, such as diagnosing primary immunodeficiency disorders, and tested only a few LLMs, such as GPT-4o and LLama, lacking evaluation of recently released LLMs like DeepSeek and Qwen.

Rheumatology and clinical immunology encompass numerous disease types with complex pathogenesis and highly specialized diagnosis and treatment plans. Evaluating LLMs' professional capabilities in this field can provide a basis for medical workers, educators, and researchers to select appropriate AI-assisted tools. Also, it helps identify current models' weaknesses in specialized knowledge understanding and clinical application, providing direction for subsequent domain adaptation fine-tuning. This paper is the first attempt to comprehensively evaluate LLMs' professional knowledge capabilities in immunology, an important medical field. We select the latest representative LLMs, such as GPT-4.1, DeepSeek-R1, Gemma3, and Qwen3, as test subjects, conducting large-scale testing experiments. We purchasesimulated questions from reputable online vendors that mimic the format and content of the Chinese Health Professional Technical Qualification Examination in Rheumatology and Immunology for testing. These questions cover basic medical knowledge, immunology-related medical professional knowledge, immunology professional knowledge, and professional practice ability. The test results show that DeepSeek-R1 and Qwen3 demonstrate strong capabilities in the evaluation of rheumatology and clinical immunology professional knowledge, especially in basic knowledge and professional knowledge, with accuracy stably above 90%, surpassing GPT-4.1 and Gemma3. However, these LLMs generally exhibit lower accuracy in handling professional practice ability questions, revealing limitations in complex clinical scenario applications. This study provides methodological and empirical references for evaluating LLMs' knowledge mastery and application abilities in specialized medical fields and points out directions for future model optimization and evaluation system improvement.

## 2 Methods

### 2.1 Dataset

The dataset used for evaluation in this study consists of simulated questions purchased from online vendors that are designed to reflect the format and content of the Rheumatology and Clinical Immunology subject of the Chinese Health Professional Qualification Examination. All these questions are original Chinese questions. This exam is a national-level access examination for health technicians engaged in rheumatology and clinical immunology professional work in China, serving as a standard examination to assess whether applicants possess the corresponding professional technical qualifications and abilities (23). This technical qualification examination aims to scientifically and fairly measure and evaluate medical personnel's professional knowledge, professional skills, and clinical practice abilities in the field of immunology, ensuring that medical personnel engaged in this specialty meet uniform, standardized basic levels, guaranteeing medical quality and patient safety.

The question bank content used in this paper is comprehensive, covering the core knowledge and skills required for rheumatology and clinical immunology physicians. All questions are single-choice and divided into four categories: Basic Medical Knowledge, Immunology-Related Medical Professional Knowledge, Immunology Professional Knowledge, and Professional Practice Ability, totaling 2829 questions. The Basic Medical Knowledge category comprises 517 questions, covering basic medical disciplines such as anatomy, physiology, pathology, and pharmacology related to rheumatology and immunology. The Immunology-Related Medical Professional Knowledge category includes 1698 questions, involving knowledge closely related to the diagnosis and treatment of rheumatic and immunological diseases in related disciplines such as internal medicine, surgery, pediatrics, and dermatology. The Immunology Professional Knowledge category consists of 570 questions, focusing on core professional knowledge such as immune system fundamentals, autoimmune disease mechanisms, and immunological testing. The Professional Practice Ability category includes 44 questions, simulating clinical scenarios to assess comprehensive application abilities such as medical history analysis, auxiliary examination interpretation, diagnosis, and treatment plan selection.

Below is an example question from the Immunology Professional Knowledge category:

**Question**: Which of the following corresponds to the pathological features of dry syndrome affecting the lip mucosa? ( )

A. Angioma formation

B. Destruction of glandular tissue, focal lymphocytic infiltration

C. Leukocytosis

D. Complement deposition

E. NK cell infiltration

**Answer**: B

### 2.2 Tested models

To ensure a comprehensive evaluation of LLMs with different architectures and scales, this study includes a wide variety of models in the experiments. The selected LLMs include both large cutting-edge models that perform excellently in natural language processing tasks and small lightweight models deployable in resource-constrained environments. Considering cost and performance, we select the following representative model families: the DeepSeek series, OpenAI's GPT series, Google's Gemma series, and Alibaba's Qwen series, totaling 11 LLMs, as detailed in Table 1.

**Table 1:** List of tested LLMs.

**No**.	**Model**	**Parameters**	**Release date**	**Company**	**API service**
1	DeepSeek-R1	671B	January 20, 2025	DeepSeek	Silicon flow
2	DeepSeek-R1-32B	32B	January 20, 2025	DeepSeek	Silicon flow
3	DeepSeek-v3(pro)	671B	March 24, 2025	DeepSeek	Silicon flow
4	GPT-4o	200B	May 13, 2024	OpenAI	OpenAI
5	GPT-4.1	N/A	April 15, 2025	OpenAI	OpenAI
6	GLM4-32B	32B	April 14, 2025	THUDM	Silicon flow
7	Llama4-scout	109B	April 5, 2025	Meta	LlmAPI
8	Gemma2-27B	27B	June 28, 2024	Google	Silicon flow
9	Gemma3-27B	27B	March 12, 2025	Google	LlmAPI
10	Qwen3-32B	32B	April, 29,2025	Alibaba	Silicon flow
11	Qwen3-235B	235B	April 29, 2025	Alibaba	Silicon flow

### 2.3 Testing methods

This study employs a unified automated testing process to evaluate all participating LLMs. During testing, programmatic calls are made through each model's provided API interface. Automated test scripts written in Python read question and option information line by line from the Excel-format question bank file. To ensure methodological consistency and fair comparison across heterogeneous LLMs, all models are evaluated using a zero-shot prompting strategy. This avoids introducing model-specific biases related to system prompts, hidden instructions, or chain-of-thought behavior, which differ substantially among providers. Zero-shot evaluation reflects the default usage patterns in many real-world clinical and educational settings, where users typically input single-turn questions without specialized prompting. We therefore designed the evaluation to measure baseline reliability and factual correctness under uniform and controlled conditions.

To ensure comparability of evaluation results and reduce the impact of random factors, all LLMs use default parameter settings without any system prompts or chain-of-thought guidance to examine the models' native capabilities under default configurations. Note that all questions used in this study are original Chinese questions. No translation is performed at any stage of data preparation or model evaluation. All prompts given to the models were also in Chinese. This ensures consistency with the linguistic structure of the original exam and avoids any biases introduced by translation.

## 3 Results

**Figure 1:**
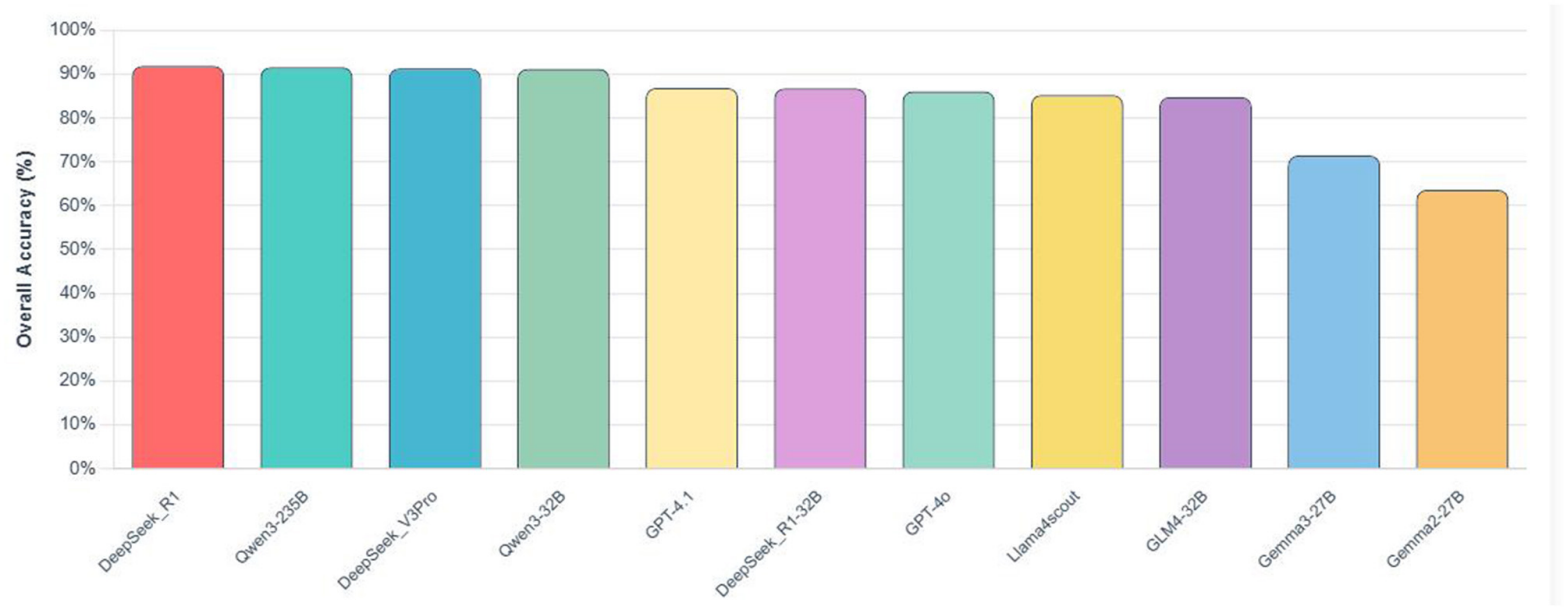
Total accuracy of tested LLMs.

Based on the test results from question bank, the performance of the 11 LLMs across the four knowledge dimensions is shown in Table 2, with total accuracy shown in Figure 1. Overall, the LLMs show significant differences in performance, with total accuracy rates ranging from 63.63% to 91.91%.

**Table 2:** Test results (I: basic medical knowledge; II: related medical professional knowledge; III: immunology professional knowledge; IV: professional practice ability).

**LLMs**	**Test I**	**Test II**	**Test III**	**Test IV**	**Total accuracy**
DeepSeek-R1	94.20%	92.58%	88.91%	81.82%	91.91%
DeepSeek-R1-32B	88.59%	88.67%	80.70%	77.27%	86.78%
DeepSeek-V3Pro	93.23%	92.46%	87.52%	81.82%	91.34%
GPT-4.1	86.46%	87.10%	86.84%	84.09%	86.89%
GPT-4o	86.65%	86.75%	84.39%	77.27%	86.11%
Llama4scout	84.53%	86.75%	82.63%	72.73%	85.30%
Gemma2-27B	63.25%	63.25%	65.78%	54.54%	63.63%
Gemma3-27B	69.44%	71.60%	73.16%	70.45%	71.47%
GLM4-32B	87.23%	86.22%	79.12%	77.27%	84.84%
Qwen3-235B	91.68%	93.46%	87.19%	79.07%	91.62%
Qwen3-32B	93.20%	92.79%	87.28%	84.09%	91.23%

In the Basic Medical Knowledge section (517 questions), DeepSeek_R1 rankes first with 94.2% accuracy, followed by Qwen3-32B (93.2%) and DeepSeek_V3Pro (93.23%). Gemma2-27B performed relatively weakly in this dimension, with an accuracy of 63.25%. In the Related Medical Professional Knowledge section (1698 questions), Qwen3-235B performs excellently, achieving 93.46% accuracy, with DeepSeek_R1 (92.58%) and Qwen3-32B (92.79%) ranking second and third, respectively. In the Immunology Professional Knowledge section (570 questions), DeepSeek_R1 leds with 88.91% accuracy, while Qwen3-32B (87.28%) and DeepSeek_V3Pro (87.52%) also perform excellently. In the Professional Practice Ability section (44 questions), GPT-4.1 and Qwen3-32B tie for the highest accuracy, both at 84.09%, followed by DeepSeek_R1 and DeepSeek_V3Pro with 81.82% accuracy.

In terms of total accuracy, as shown in Figure 1, DeepSeek_R1 rankes first with a comprehensive performance of 91.91%, followed by DeepSeek_V3Pro (91.34%) and Qwen3-235B (91.62%) in second and third place, respectively. Qwen3-32B rankes fourth with a total accuracy of 91.23%. In contrast, the Gemma series models perform relatively poorly overall, with Gemma2-27B achieving 63.63% total accuracy and Gemma3-27B achieving 71.47%. To further verify the reproducibility of our findings, we conduct three independent runs for four representative models (DeepSeek-R1, GPT-4.1, Gemma3-27B and Qwen3-235B) using the same zero-shot setting. Across 570 items in the Immunology Professional Knowledge section, the per-run accuracy varied by less than 1.2 percentage points for all four models indicating deterministic behavio r under deterministic decoding and confirming that the reported point estimates are highly stable across replicate calls.

To systematically evaluate performance differences among 11 LLMs, we conduct pairwise McNemar tests on 570 specialized knowledge questions. Results show that any pairwise comparison within DeepSeek-R1, Qwen3-235B, and Qwen3-32B yielded *p*≥0.62, indicating comparable performance with differences attributable to random variation. However, these three top performers exhibited *p* < 0.001 against GPT-4o, GPT-4.1, Llama4-scout, and the Gemma series, statistically forming a significant high-to-medium stratification. Concurrently, p-values between any models within the DeepSeek family or Gemma family remained no less than 0.62, suggesting that different checkpoint sizes within the same series converge in performance on this dataset.

## 4 Discussion

### 4.1 Domain-specific interpretation of LLM performance

The performance of the 11 evaluated LLMs demonstrates clear differences across the four major competency domains of the examination. These patterns reveal not only the relative strengths of current models in factual medical knowledge but also their limitations in deeper clinical reasoning. Below, we provide a domain-by-domain interpretation to highlight the cognitive and practical implications of the results.

Firstly, within the domain of fundamental medical knowledge, most high-performance large language models (LLMs) achieve accuracy rates exceeding 90%, with models such as DeepSeek-R1 and Qwen3-32B demonstrating mastery approaching expert-level proficiency. This dimension primarily assesses structured medical knowledge, content which is comprehensively represented within mainstream pre-training corpora. Test results indicate that large language models excel in pattern-based semantic retrieval and factual recall, cognitive processes corresponding to the early stages of human medical learning. These findings confirm that contemporary large language models have established a robust and reliable foundation of medical knowledge.

Second, in the Related Medical Professional Knowledge domain, which spans internal medicine, dermatology, pediatrics, and other adjacent clinical specialties, performance remained strong for most large-scale models. This domain requires broader semantic integration across multiple disciplines, yet the knowledge involved is still largely factual and well documented. The moderate performance differences among models suggest that large, diverse pretraining corpora enable effective generalization across medical subfields. However, the more pronounced decline observed in mid-sized models such as Gemma2-27B highlights the dependence of cross-disciplinary reasoning on model scale and training data diversity.

Third, performance in the Immunology Professional Knowledge domain displayed greater variability across models. Although leading models scored around 86-89%, overall accuracy was lower than in the previous two domains. Questions in this category demand understanding of mechanisms such as immune pathways, autoimmunity, and immunological testing–areas where high-quality textual resources are more specialized and less abundant. This type of knowledge requires deeper conceptual abstraction and mechanistic understanding, capabilities that current LLMs approximate but do not fully master. The results underscore the need for immunology-specific domain adaptation or fine-tuning to improve performance in specialized medical subfields.

Fourth, the domain of professional practice capability reveals the most significant limitations of current large language models. Even the best-performing models, GPT-4.1 and Qwen3-32B, achieve accuracy rates of only around 84%, with other models performing markedly worse. These test items simulate real clinical scenarios, requiring diagnostic reasoning based on symptoms and examination results. Unlike factual questions, such tasks demand multi-step reasoning, situational judgement, and probabilistic inference. The performance gap reflects the challenges LLMs face when executing evidence-based clinical reasoning. Despite possessing robust factual knowledge reserves, current models remain constrained when handling complex, context-dependent medical tasks.

### 4.2 Implications for medical education and assessment

The domain-specific performance patterns observed in this study carry several important implications for medical education and assessment. The strong results in factual and foundational knowledge indicate that advanced LLMs can serve as supportive tools for medical learning, helping generate practice questions, clarify complex immunology concepts, and provide structured summaries that ease the acquisition of basic content. However, the marked decline in the Professional Practice Ability domain highlights that core clinical reasoning, such as differential diagnosis, contextual judgment, and multi-step inference, remains a distinctly human strength and should continue to be emphasized in student evaluation and curriculum design. These results suggest that while LLMs can enhance early-stage knowledge acquisition, high-stakes assessment must still rely on tasks that probe deeper cognitive processes. At the same time, the domain-specific strengths and weaknesses revealed in this study provide a basis for developing targeted AI-assisted tutoring systems that reinforce foundational knowledge while offering guided reasoning scaffolds for complex clinical scenarios, ultimately supporting medical students without substituting expert oversight.

### 4.3 Technical factors, clinical implications, and future directions

Differences in model performance can be partly explained by variations in model architecture, pretraining corpus diversity, and domain exposure. Larger models such as DeepSeek-R1 and Qwen3-235B likely benefit from extensive medical coverage and richer Chinese pre-training data, contributing to their strong performance across most domains. In contrast, mid-sized models like Gemma2-27B and Gemma3-27B may lack sufficient medical domain representation during pretraining, resulting in weaker performance, especially in tasks requiring immunology-specific conceptual knowledge or integrated clinical reasoning. The absence of explicit domain fine-tuning in all evaluated models also helps explain the consistent decline observed in tasks involving higher-order pathophysiological interpretation.

The findings additionally highlight important clinical and methodological implications. Although the leading LLMs show impressive mastery of factual medical knowledge, their reduced accuracy in practice-oriented items indicates that autonomous diagnostic reasoning remains beyond their current capabilities. As such, LLMs may serve as educational support tools but should not be deployed for unsupervised clinical decision-making. The use of single-choice questions and zero-shot settings in this study, while reflecting common real-world usage, limits the evaluation of deeper reasoning processes and fails to capture the full complexity of clinical workflows.

Future research should therefore broaden the evaluation framework to include open-ended clinical reasoning tasks, longitudinal case simulations, and multimodal clinical data such as imaging or laboratory results. Incorporating chain-of-thought prompting, domain-specific fine-tuning, and reinforcement learning with expert feedback may further enhance reasoning stability and domain robustness. Comparative analyses using real patient cases and collaborative expert annotation will also be essential for understanding how LLMs behave in authentic clinical environments and for identifying safe, practical pathways for their integration into medical education and clinical decision support systems.

## 5 Conclusion

This study systematically evaluated the professional capabilities of 11 representative LLMs in the field of rheumatology and clinical immunology. The results show that the DeepSeek series and Qwen series models performed excellently in this professional field, with total accuracy rates exceeding 91%, demonstrating their significant advantages in mastering medical professional knowledge. However, all models showed decreased performance on questions examining comprehensive clinical practice ability, highlighting the limitations of LLMs in complex clinical reasoning.

The findings suggest that LLM performance is shaped by multiple factors, including model scale, training data composition, architectural design, and the degree of domain adaptation. While leading models now approach the knowledge reserve levels of human experts in basic and related medical fields, their constrained clinical reasoning indicates that current LLMs are better suited for roles in medical education and knowledge support rather than independent clinical decision-making. Future research should prioritize the development of more advanced and multifaceted evaluation frameworks, such as incorporating multimodal inputs, integrating simulations of authentic clinical decision pathways, and conducting deeper error analyses in collaboration with clinical immunology specialists. Additionally, domain-adapted fine-tuning and targeted enhancement of reasoning capabilities represent essential directions for improving the reliability and practical applicability of LLMs in clinical environments.

## Data Availability

The original contributions presented in the study are included in the article/supplementary material, further inquiries can be directed to the corresponding author.
